# Fisetin protects against cardiac cell death through reduction of ROS production and caspases activity

**DOI:** 10.1038/s41598-020-59894-4

**Published:** 2020-02-19

**Authors:** Sophie Rodius, Niek de Klein, Céline Jeanty, Héctor Sánchez-Iranzo, Isaac Crespo, Mark Ibberson, Ioannis Xenarios, Gunnar Dittmar, Nadia Mercader, Simone P. Niclou, Francisco Azuaje

**Affiliations:** 1 Quantitative Biology Unit, Luxembourg Institute of Health (LIH), Luxembourg, 1445 Strassen Luxembourg; 2 Department of Genetics, University of Groningen, Groningen, 9700 RB The Netherlands; 3 The author completed this work at the Department of Oncology, Luxembourg Institute of Health (LIH), Luxembourg, 1445 Strassen Luxembourg; 4 Development of the Epicardium and Its Role During Regeneration Group, Centro Nacional de Investigaciones Cardiovasculares Carlos III, 28029 Madrid, Spain; 5 Vital-IT Systems Biology Division, SIB Swiss Institute of Bioinformatics, Lausanne, CH-1015 Switzerland; 6 Center for Integrative Genomics, University of Lausanne, Lausanne, CH-1015 Switzerland; 7 Department of Biochemistry, University of Geneva, 1211 Geneva, Switzerland; 8 Institute of Anatomy, University of Bern, Bern, Switzerland; 9 Department of Oncology, Luxembourg Institute of Health (LIH), Luxembourg, 1445 Strassen, Luxembourg; 10 Present Address: Current affiliation: Data and Translational Sciences, UCB Celltech, 208 Bath Road, Slough, SL1 3WE United Kingdom

**Keywords:** Apoptosis, Data mining, Phenotypic screening, Heart failure

## Abstract

Myocardial infarction (MI) is a leading cause of death worldwide. Reperfusion is considered as an optimal therapy following cardiac ischemia. However, the promotion of a rapid elevation of O_2_ levels in ischemic cells produces high amounts of reactive oxygen species (ROS) leading to myocardial tissue injury. This phenomenon is called ischemia reperfusion injury (IRI). We aimed at identifying new and effective compounds to treat MI and minimize IRI. We previously studied heart regeneration following myocardial injury in zebrafish and described each step of the regeneration process, from the day of injury until complete recovery, in terms of transcriptional responses. Here, we mined the data and performed a deep *in silico* analysis to identify drugs highly likely to induce cardiac regeneration. Fisetin was identified as the top candidate. We validated its effects in an *in vitro* model of MI/IRI in mammalian cardiac cells. Fisetin enhances viability of rat cardiomyocytes following hypoxia/starvation – reoxygenation. It inhibits apoptosis, decreases ROS generation and caspase activation and protects from DNA damage. Interestingly, fisetin also activates genes involved in cell proliferation. Fisetin is thus a highly promising candidate drug with clinical potential to protect from ischemic damage following MI and to overcome IRI.

## Introduction

Myocardial infarction (MI), the most common ischemic heart disease, is a leading cause of death worldwide^1^. It is a multifactorial disease: besides genetic predispositions, other parameters, e.g., smoking and obesity, are considered as prominent risk factors^2^. MI is most often caused by the blockade of a coronary artery after the rupture of an atherosclerotic plaque. This obstruction prevents blood supply (ischemia) to a part of the heart. Consequently, heart cells normally supplied with oxygen and nutrients by the impacted coronary artery die and are replaced by a fibrotic scar. While formation of this scar is crucial to prevent the rupture of the ventricular wall, excessive fibrotic response and reactive fibrosis in the uninjured myocardium can ultimately lead to left ventricular dysfunction, heart failure and death^3^.

During MI, myocardial cells are deprived from oxygen (hypoxia) and nutrients (starvation), leading to cell death. Apoptosis appears to play a predominant role in cardiomyocyte loss after MI, both in the infarcted and peri-infarcted myocardium. It determines infarct size, degree of left ventricular remodeling and onset of heart failure^4,5^. Two main apoptotic pathways can be observed: the intrinsic or mitochondrial pathway, and the extrinsic or death receptor pathway. Both are linked, can influence each other and converge on the same terminal signalling resulting in DNA fragmentation, protein degradation, formation of apoptotic bodies and ingestion by phagocytic cells^6^. The extrinsic apoptotic pathway is activated by extracellular signals *via* ligands interacting with transmembrane death receptors, members of the tumor necrosis factor (TNF) superfamily^7–9^. The intrinsic apoptotic pathway is induced by different stimuli, such as hypoxia, deprivation of growth factors or oxidative stress. This pathway is activated when the mitochondrial membrane, whose integrity is regulated by BCL-2 family members, is permeabilized^7,10,11^. Caspases, a family of cytoplasmic endoproteases, are key regulators of apoptosis^7^. Following MI, cells suffer both from hypoxia and deprivation of growth factors, but also from oxidative stress due to the direct decrease in O_2_ level and the generation of reactive oxygen species (ROS)^12,13^. Mitochondria are the main consumers of O_2_ and the major producers of ROS in the cell. At physiological levels, ROS modulate various cellular processes, including: hypoxic response, growth factor signaling and inflammation. However, at higher concentration, ROS promote damages in DNA, proteins and membrane lipids, i.e., oxidative stress. Consequently, the cellular level of ROS must be highly regulated by the antioxidative capacity of the cell. In response to hypoxia, mitochondria modify their metabolism through modification of their respiratory chain and activation of hypoxia inducible factors (HIF) to keep ROS at relatively low levels^13–15^.

MI therapies aim at quickly restoring the blood flow to the heart, using either medications to dissolve the thrombotic clot or surgery, e.g., percutaneous coronary intervention (PCI). However, although reoxygenation is crucial for patient survival, reperfusion promotes a rapid elevation in O_2_ levels in ischemic cells, which induces a high production of ROS in mitochondria resulting in cardiomyocytes injury. This phenomenon, called ischemia reperfusion injury (IRI), can lead to cardiac remodeling and heart failure^16,17^. Because of these challenges and a relatively slow progress in the development of new drugs, there is a crucial need for new effective therapies to treat MI and overcome IRI.

To meet this clinically-relevant need, we implemented here a systematic approach for drug repositioning, which tapped into an extensive collection of expression signatures obtained from treated cancer cell lines. Our hypothesis was that a drug predicted to induce, *in vitro*, expression signatures observed at different key stages of heart regeneration in the zebrafish could also represent a promising compound for cardiac damage healing and IRI reduction. We selected the top candidate compound: fisetin, a flavonoid. Flavonoids, phytonutrients present in almost all kinds of fruits and vegetables, are polyphenolic compounds that fulfill many functions in plants^18^. They have recently emerged as powerful antioxidants providing health benefits in humans, protecting from degenerative diseases linked to oxidative stress such as cancer, diabetes and cardiovascular diseases^19,20^.

This study follows up our previous findings, where we studied the different steps of heart regeneration following myocardial injury in the zebrafish^21^. We were able to elucidate the dynamic gene co-expression network associated with key stages of heart regeneration following injury. Here, based on these data and the above-stated hypothesis, we developed a new computational method to find drugs capable to induce the transcriptional patterns required for heart regeneration as observed in the zebrafish. We predicted and ranked candidate drugs, and performed experiments on mammalian cardiac cells subjected to an *in vitro* MI/IRI model to validate the effect of the top candidate drug: fisetin. Results indicate that fisetin is able to enhance cell viability of rat cardiomyocytes following hypoxia/starvation – reoxygenation, to protect from apoptotic cell death, by decreasing ROS generation as well as caspases activation, and to reduce DNA damage. These findings put forward fisetin as an excellent candidate for limiting cardiac damage due to ischemia following MI and for overcoming IRI.

## Results

### Identification of candidate compounds for repositioning through a systematic computational prediction strategy

**Figure 1:**
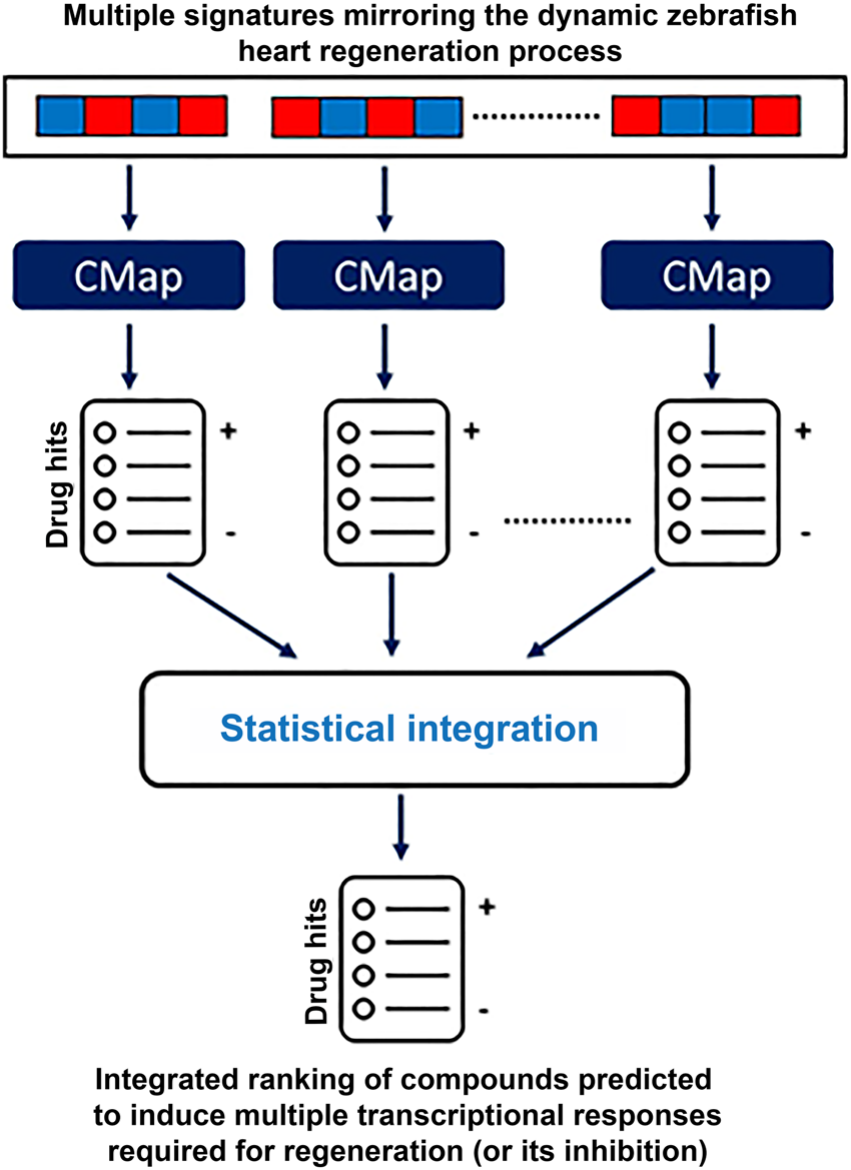
Systems-level prediction of candidate drugs with potential to induce transcriptional responses as observed *in vivo* during heart regeneration in the zebrafish. We predicted candidate compounds for repositioning based on a new computational strategy that matches multiple heart regeneration expression signatures with drug-induced expression profiles in the Connectivity Map (CMap).

We generated an integrated, statistically-ranked list of compounds positively (or negatively) matched to the expression signatures identified in our heart regeneration gene expression dataset (Fig. 1, Methods). Our strategy is based on the exact estimation of probability distributions of rank product statistics. In our project, such signatures represented the dynamic transcriptional changes observed at each time point during the regeneration process as compared to 4 hours after injury: 5 time points, from 1 day to 90 days post-injury. We analyzed hundreds of gene expression signatures potentially relevant and sufficiently robust to mirror salient molecular states associated with the regeneration process. Based on a combination of computational processing and human expert analysis, we selected different sets of most relevant dynamic signatures for different post-injury (and reference) time points. The size of these signatures ranged from a few dozen to hundreds of genes, and they were significantly enriched in diverse biological processes relevant to cardiac regeneration. Next, we focused on a set of 7 signatures that accurately and meaningfully represented key stages of the regeneration process. They included, for example, a signature representing expression changes at 1 day post-injury (in relation to 4 hrs post-injury).

We identified, ranked and curated a list of candidate compounds for repositioning. Such compounds were predicted to either induce or inhibit transcriptional programs that closely resemble those observed during the zebrafish regeneration process. This resulted in the identification of fisetin as the top repositioning candidate (integrated p-value = 2.17E-11). We also identified withaferin A, a compound with diverse clinical applications including in cancer^22^, as negatively connected to cardiac cell regeneration in the zebrafish (integrated p-value = 1.80E-09). Thus, withaferin was predicted as a compound that is detrimental to cardiac cell regeneration by inducing, unlike fisetin, transcriptional signatures negatively correlated with the expression signatures observed during heart regeneration. Full list of the predicted positive and negative candidate drugs are available on https://lih-biomod.lu/infused/.

### Fisetin enhances viability of rat H9c2 cardiomyocytes following hypoxia/starvation - reoxygenation

**Figure 2:**
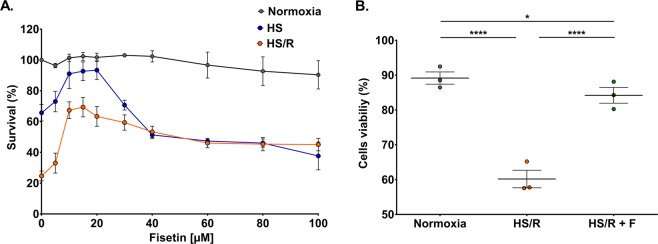
Fisetin enhanced the viability of H9c2 cardiomyocytes. (**A**) Survival tests were performed in normoxia, HS or HS/R experiments. Different concentrations of fisetin (from 0 to 100 μM) were tested. In normoxia experiments cells were cultured during 24 h in normal conditions, while in HS experiments cells were cultured in serum free DMEM at 0.5% O_2_, both in presence of the drug. In the HS/R experiments, cells were treated with fisetin following HS, during the whole reoxygenation phase. Cell survival was monitored by CyQuant assay. Results are expressed as the mean of three independent experiments performed in triplicate (error bars: SEM). (**B**) Effect of fisetin on cardiomyocytes viability upon HS/R treatment. Cell viability was measured in FACS by annexin V/PI staining: annexin V−/PI− cells were considered as viable. Normoxia: cells cultured under normoxia without fisetin (control group). HS/R: cells subjected to HS/R, treated with DMSO as vehicle control. HS/R + F: cells subjected to HS/R, treated with 15 μM fisetin. Results are expressed as the mean of three independent experiments. One-way ANOVA. Post-hoc analysis by Tukey. *P ≤ 0.05, ****P ≤ 0.0001.

In order to study the effect of fisetin on cell survival, we tested ten different concentrations of the drug (0, 5, 10, 15, 20, 30, 40, 60, 80 and 100 μM) on H9c2 cardiomyocytes. Cells were cultured under normoxia, in hypoxia/starvation (HS) or subjected to hypoxia/starvation – reoxygenation (HS/R)^23,24^. In each experiment, fisetin was applied during 24 h before cell analysis (see Methods). Cell viability was assessed by CyQuant Assay (Fig. 2A). Fisetin showed no sign of cell toxicity, even at the highest concentration (100 μM) and didn’t affect the viability of cardiomyocytes cultured in normoxia. Compared to cells cultured in normoxia, HS/R and HS caused a decrease of 75% and 40% in cell survival, respectively. Fisetin significantly increased cell survival, with a peak at 15 μM where the drug restored cell viability by up to 70% for cells subjected to HS/R. For cells cultured in HS, the peak in viability was at 20 μM with 90% of cell survival. On the contrary, withaferin A, decreased cell survival in a concentration-dependent manner, with an IC_50_ of 2.6 μM (Data not shown). DMSO, used as drug vehicle, didn’t affect cell viability. These results indicate that fisetin markedly enhanced survival of H9c2 cardiomyocytes following HS or HS/R. We focused our work on HS/R, our *in vitro* MI/IRI model. We therefore used the most effective concentration of 15 μM of fisetin in the subsequent experiments. We first validated the effect of fisetin on cell viability by FACS. H9c2 cardiomyocytes were cultured in serum free medium at 0.5%0_2_ during 24 h to mimic hypoxia/starvation, then during 24 h in normoxic conditions in complete medium to mimic reoxygenation. When indicated (HS/R + F) fisetin was applied during the whole reoxygenation phase. As expected, 15 μM of fisetin significantly reduced the cytotoxic effect of HS/R treatment (Fig. 2B).

### Fisetin protects H9c2 cardiomyocytes from apoptotic cell death

**Figure 3:**
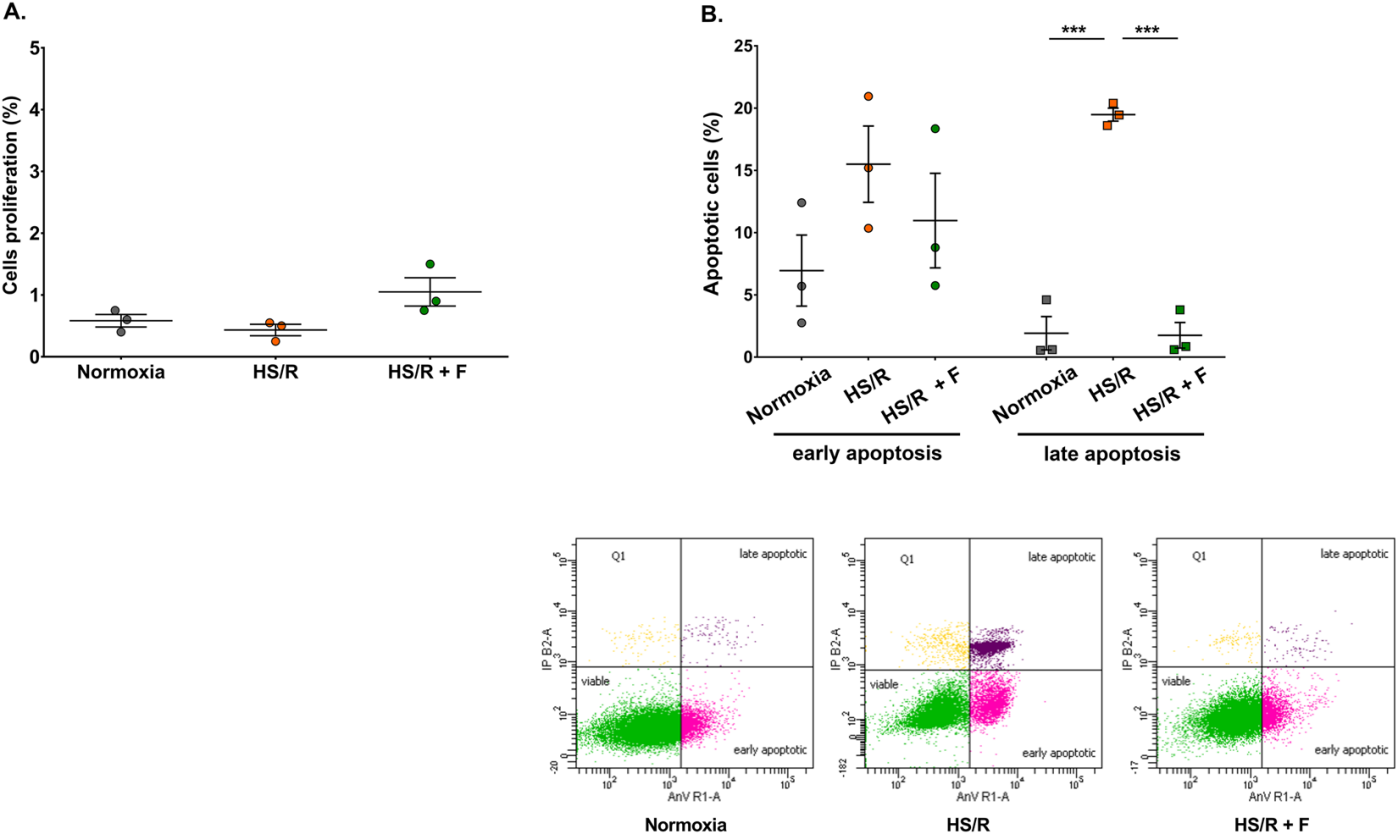
Effect of fisetin on cardiomyocytes proliferation and apoptosis. Flow cytometry experiments were carried out to measure the amount of proliferating and apoptotic cells in each experimental group. (**A**) Proliferation of H9c2 cells assessed by Ki67 staining. Ki67+ cells are proliferating. Results are expressed as the mean of three independent experiments performed in triplicate. One-way ANOVA. Post-hoc analysis by Tukey. No significant difference: P > 0.05. (**B**) Dot plot representation of H9c2 apoptotic ratio measured by annexin V/PI staining. Annexin V−/PI− cells were considered as viable, annexin V+/PI− cells were considered as early apoptotic and annexin V+/PI+ cells were considered as late apoptotic. Normoxia: control group, cells cultured under normoxia. HS/R: cells subjected to HS/R, treated with DMSO as vehicle control. HS/R + F: cells subjected to HS/R, treated with 15 μM fisetin. Results are expressed as the mean of three independent experiments performed in triplicate. Two-way ANOVA. Post-hoc analysis by Tukey. ***P ≤ 0.001.

In our experiments, survival/viability was measured by CyQuant Direct assay, based on estimation of DNA content of healthy cells which can be directly correlated with cell number, or by annexin V/PI staining which labels apoptotic cells and allows to determine the number of remaining healthy cells in a given population. Since fisetin affected cell survival/viability, i.e. cell numbers, we studied more precisely the effects of the drug on H9c2 cardiomyocytes proliferation and apoptosis following HS/R. Fisetin markedly increased proliferation of neonatal rat cardiomyocytes (2 fold) measured by Ki67 staining (Fig. 3A), a result confirmed by cell cycle analysis showing a higher number of cells in S phase following fisetin treatment (Data not shown). The effect of the drug - increase in proliferation of 0.6% - was however not statistically significant and too low to impact the global number of cells and explain the results obtained in the survival/viability experiments. In order to investigate whether fisetin was able to protect cardiomyocytes from HS/R induced cell death, we performed an annexin V/PI staining. Annexin V−/PI− cells were considered as viable cells, while annexin V+/PI− cells were considered as early apoptotic and annexin V+/PI+ cells as late apoptotic (Fig. 3B). HS/R significantly increased apoptotic cell death compared to cells cultured in normoxia (from 6.9% to 15.5% for early apoptosis, and from 1.9% to 19.5% for late apoptosis, P ≤ 0.001). In the presence of fisetin, cell apoptosis in HS/R was markedly reduced: from 15.5% to 11% for early apoptosis and from 19.5% to 1.7% for late apoptosis (P ≤ 0.001). These results clearly show the protective effect of fisetin against HS/R-induced cardiomyocyte death.

### Fisetin regulates expression of genes related to cardioprotection and to cardiomyocytes proliferation and maturation

**Figure 4:**
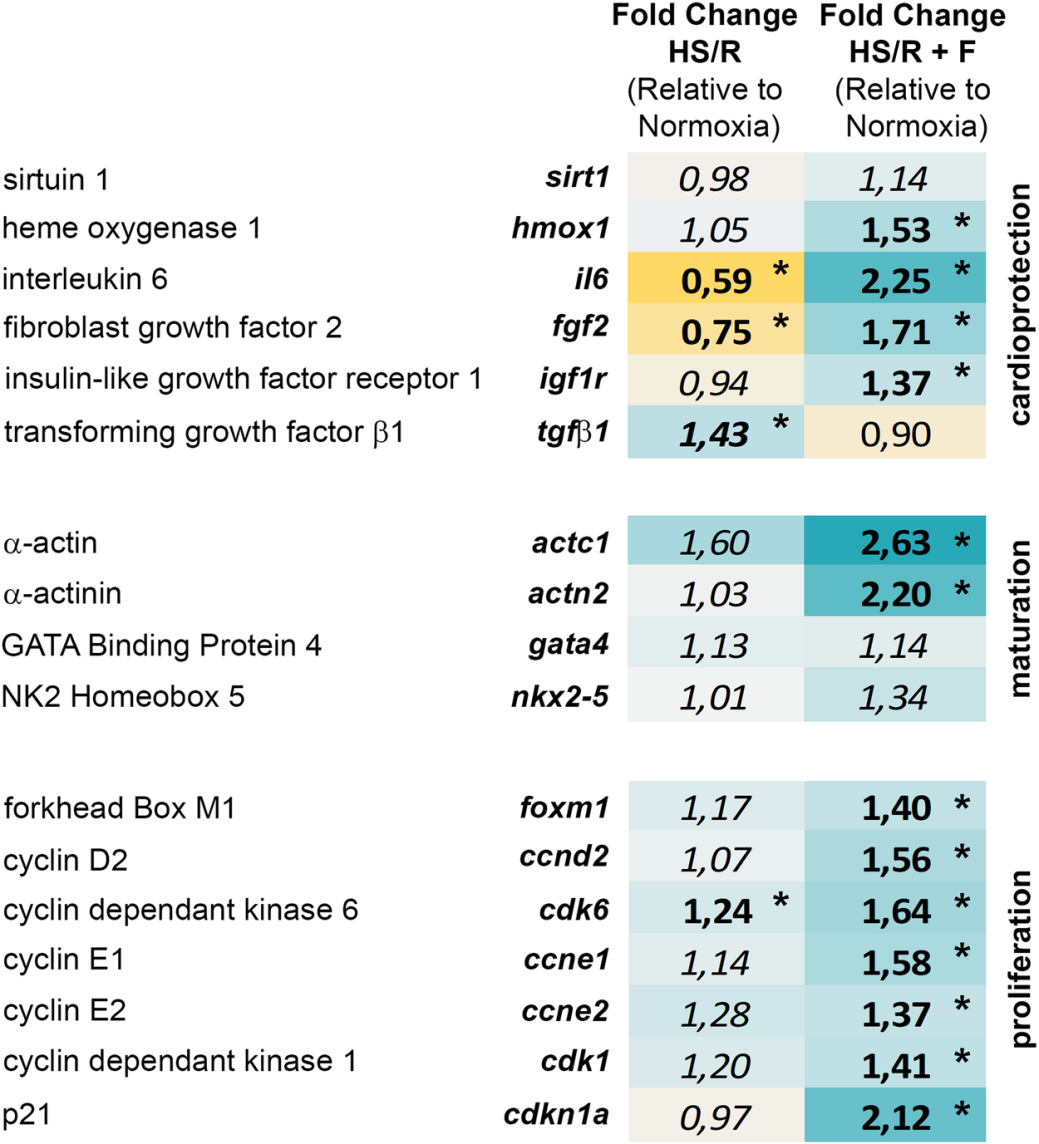
Fisetin regulated a panel of genes related to cardioprotection, proliferation and maturation. Gene expression was assessed by qRT-PCR in cells cultured under normoxia, subjected to HS/R and treated with DMSO as vehicle control (HS/R) or subjected to HS/R and treated with 15 μM fisetin (HS/R + F) during 24 h. Results are expressed as the mean of four independent experiments. Statistical significance was determined using a one-way ANOVA corrected for multiple testing with a Tukey-Kramer as post-test (corrected p-value < 0.05). Statistically significant results are indicated in bold with a star. Fold change < 1: genes down-regulated compared to normoxia (yellow). Fold change > 1: genes up-regulated compared to normoxia (blue).

We studied the effect of fisetin on cardioprotection, cardiomyocyte proliferation and cardiomyocyte maturation by qRT-PCR (Fig. 4). Of note, while we couldn’t detect any noticeable increase in *sirt*1 (sirtuin 1, a known fisetin target^25^) expression in our experimental model, we observed a significant up-regulation of four cardioprotective genes following fisetin treatment: *hmox1* (heme oxygenase 1), *il6* (interleukin 6, another known fisetin target^26,27^), *fgf*2^28,29^ (fibroblast growth factor 2) and *igf1r* (insulin-like growth factor receptor 1). Moreover, while inhibition of the TGFβ1 pathway is known to protect H9c2 cardiomyocytes from IRI^30–35^, fisetin markedly decreased *tgfβ1* expression. Interestingly, while the observed increase in Ki67^+^ cardiomyocytes did not reach statistical significance (Fig. 3A), we observed an increase in the expression of genes involved in cell proliferation including *foxm1* (Forkhead Box M1)*, ccnd2* (cyclin D2), *cdk6 (*cyclin-dependent kinase 6), *ccne1* and *2* (cyclin E), as well as *cdk1* (cyclin-dependent kinase 1), whose activity is required for the cell cycle G1/S transition. This could explain the increased number of cardiomyocytes during the S phase of the cell cycle following fisetin treatment. Intriguingly, *cdkn1a* (p21) was highly up-regulated by fisetin in our experiments. While this result may seem in contradiction with the apparent activation of the above mentioned G1/S transition, it is possibly related to the protective role of p21 against apoptosis and DNA damage^36,37^. Indeed, *foxm1* was also increased, and was recently shown to be a key regulator in DNA repair^38^. Finally, fisetin seemed to promote cardiomyocytes maturation, as indicated by the up-regulation of the cardiac muscle markers *actc1* (α-actin) and *actn2* (α-actinin), while elevation of cardiac progenitor markers like *gata4* (GATA Binding Protein 4) and *nkx2–5* (NK2 Homeobox 5) were either not detectable or not significant, respectively.

### Fisetin decreases ROS production

**Figure 5:**
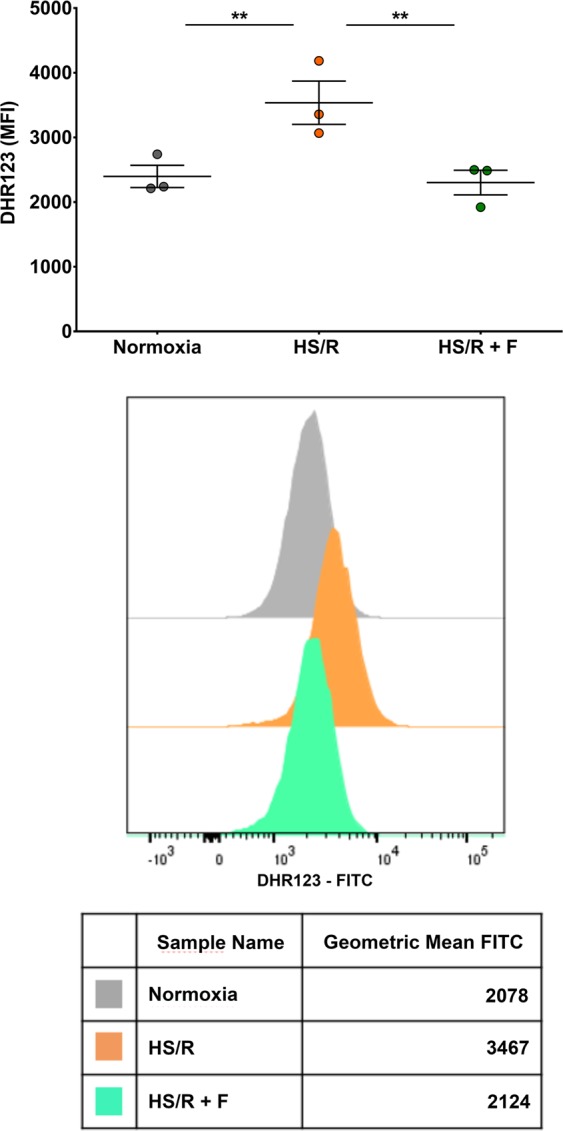
Fisetin decreased ROS expression level in cardiomyocytes. The cell permeable, nonfluorescent dihydrorhodamine 123 probe enters the cell where it is oxidized by ROS to fluorescent rhodamine 123. Fluorescence intensity, proportional to ROS expression level, was measured by flow cytometry in each experimental group. Normoxia: control group; HS/R: cells subjected to HS/R, treated with DMSO as vehicle control; HS/R + F: cells subjected to HS/R, treated with 15 μM fisetin. Results are expressed as the mean of three independent experiments. One-way ANOVA. Post-hoc analysis by Tukey. **P ≤ 0.01.

Hypoxia and reoxygenation are known to trigger an increase in ROS generation, mainly from mitochondria, leading to cell death. In our experiments fisetin protected against cell death. We therefore investigated its effect on ROS generation in H9c2 cardiomyocytes subjected to HS/R, using dihydrorhodamine 123 as ROS detection probe. ROS level was significantly elevated (1.5 fold, P ≤ 0.01) when cells were cultured in HS/R compared to cells cultured in normoxia (Fig. 5). In the presence of fisetin, ROS level in cells subjected to HS/R decreased to reach the level found in cells cultured in normoxia (P ≤ 0.01). Of note, fisetin also decreased ROS production in cardiomyocytes in HS experiments (data not shown). Altogether, fisetin prevents ROS elevation and may thus inhibit cell apoptosis by inhibiting ROS generation in cardiomyocytes subjected to HS/R.

### Fisetin inhibits caspases 8, 9 and 3 activation

**Figure 6:**
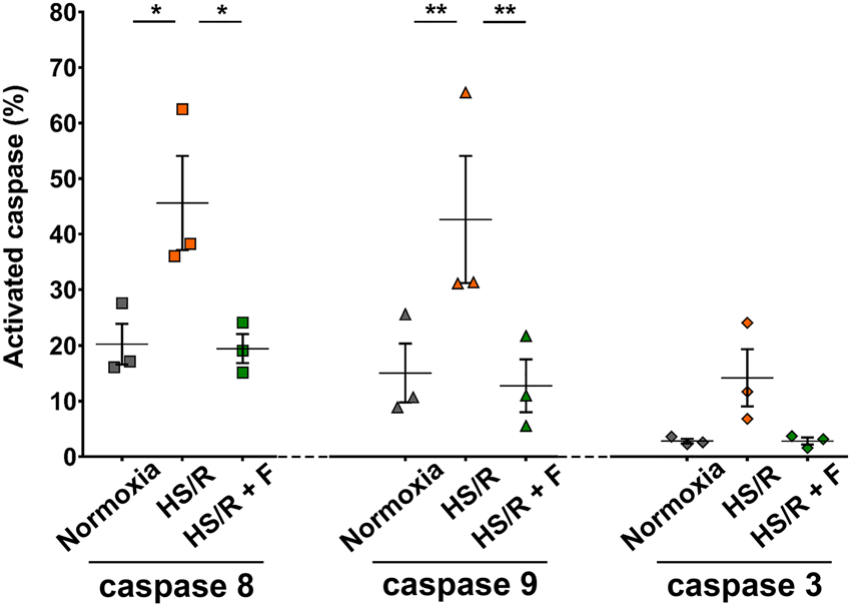
Fisetin inhibited activation of caspases 8, 9 and 3 in H9c2 cells. Caspases activity was assessed by flow cytometry using active caspase staining kits specific to caspase 8, 9 and 3, respectively. Dot plots represent the percentage of cardiomyocytes expressing activated caspase 8, 9 or 3. Normoxia: control group. HS/R: cells subjected to HS/R, treated with DMSO as vehicle control. HS/R + F: cells subjected to HS/R, treated with 15 μM fisetin. Results are expressed as the mean of three independent experiments. Two-way ANOVA. Post-hoc analysis by Tukey. **P ≤ 0.01, *P ≤ 0.05.

Caspases are key regulator enzymes of both extrinsic and intrinsic pathways of apoptosis. In order to study the putative inhibitory effect of fisetin on caspases activation, we performed caspases activity assays to measure the activity of 3 different caspases in cardiomyocytes following HS/R: caspase 8 (specific of the extrinsic apoptosis pathway), caspase 9 (activated by the mitochondrial apoptosis pathway) and caspase 3 (a final executioner caspase activated through both apoptosis pathways). The number of cells expressing activated caspases 8, 9 and 3 was highly increased when H9c2 cells were cultured in HS/R compared to cardiomyocytes cultured in normoxia (Fig. 6): 2.2 fold of increase for caspase 8 (P ≤ 0.05); 2,8 fold for caspase 9 (P ≤ 0.01) and 5 fold for caspase 3. Interestingly, fisetin was able to reverse this phenomenon, decreasing the number of caspase 8, 9 and 3 positive cells in cardiomyocytes cultured in HS/R down to the level found in cardiomyocytes cultured in normoxia. This indicates that fisetin is able to inhibit activation of caspases in both intrinsic and extrinsic apoptosis pathways in H9c2 cardiomyocytes subjected to HS/R.

### Fisetin protects from DNA damage

**Figure 7:**
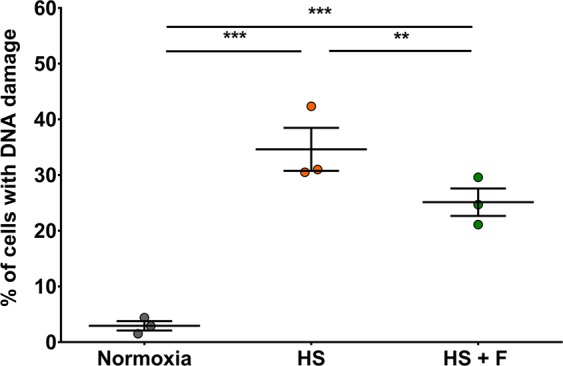
Fisetin protected cardiomyocytes cultured under HS from DNA damage. The proportion of damaged cells in each experimental group was measured by flow cytometry using the anti-8 Hydroxyguanosine antibody as DNA damage marker. Normoxia: control group. HS: cells in HS, treated with DMSO as vehicle control. HS + F: cells in HS, treated with 15 μM fisetin. Results are expressed as the mean of three independent experiments. ANOVA two factors (1: biological replicates/random; 2: Treatments). Post-hoc analysis by Tukey. ***P ≤ 0.001, **P ≤ 0.01.

Hypoxia, starvation, ROS generation and caspases activation in cells lead to DNA damage. We thus investigated whether fisetin was capable of decreasing DNA damage in cardiomyocytes subjected to HS/R, measuring 8-Hydroxyguanosine level as DNA damage marker. Intriguingly, we were not able to detect any DNA damage in cells following HS/R (data not shown). We hypothesized that cardiomyocytes may be able to repair DNA damage during the reoxygenation phase. Therefore, we aimed to detect DNA damage directly at the end of the HS period: the drug was applied during the whole hypoxia/starvation period after which cells were immediately collected and analyzed (Fig. 7). Results indicate that HS generated DNA damage in cardiomyocytes (2.9% of 8-Hydroxyguanosine positive cells when cardiomyocytes were cultured in normoxia compared to 34.6% following HS, P ≤ 0.001), while fisetin was able to reduce the proportion of damaged cells from 34.6% to 25.1% (P ≤ 0.01). Thus, fisetin shows a protective effect against DNA damage.

## Discussion

In a previous article, we studied cardiac regeneration in zebrafish using cryoinjury as MI model^21^. Unlike mammals, zebrafish has the ability to fully regenerate its heart after severe damage. The whole regeneration is tightly regulated by the dynamic interplay of multiple genes. We coupled molecular biology techniques and *in silico* modelling to analyze the dynamic transcriptional responses associated with each step of the regeneration process. In the current work, we built on such data to predict drugs likely to activate cardiac regeneration following MI.

Here, we aimed to identify novel therapeutic associations between the transcriptional profiles observed during heart regeneration in the zebrafish and those induced by drugs in several cancer cell lines. This was done through the systematic statistical integration of such datasets. Our hypothesis was that potentially relevant candidate drugs are those capable to induce gene expression profiles *in vitro* that closely resemble gene expression profiles underlying heart regeneration *in vivo*. Our multiple-signature matching pipeline predicted fisetin, a flavonoid, as the top candidate.

Flavonoids recently appeared to be powerful antioxidants. This antioxidant effect is mediated through different mechanisms such as inhibition of enzymes involved in ROS generation, ROS scavenging, chelation of metal ions implicated in generation of free radicals or activation of antioxidant defenses^18,19^. Previous studies indicate that flavonoids offer protective effects against both the initiation and the progression of atherosclerosis, which is one of the leading causes of MI^39,40^. They inhibit a key mechanism in the development of atherosclerosis: the oxidation of low density lipoproteins (LDL). Moreover, they protect nitric oxide (NO) from oxidation and increase its bioavailability, which helps restoring vascular endothelial function. Flavonoids also reduce inflammation by modulating pro-inflammatory cytokines like TNFα, decrease the expression of vascular cell adhesion molecules limiting thereby the recruitment of inflammatory cells to the arterial wall, inhibit platelets aggregation and enhance vaso-relaxation^19,41–44^. Based on their chemical structure, flavonoids can be divided in different subclasses^45,46^. Fisetin, a member of the flavonols family^43,47^, provides different beneficial effects through its anti-inflammatory^48^, antioxidant^49^, hypoglycemic^50^ or anti-obesity^51^ action. It is also known to have strong anti-cancer activity by inhibiting angiogenesis^52^, promoting cell cycle arrest in G_2_/M phase and by inducing apoptosis in cancer cells^53,54^. Moreover, it protects from coronary artery diseases via inhibition of atherosclerosis^55^.

**Figure 8:**
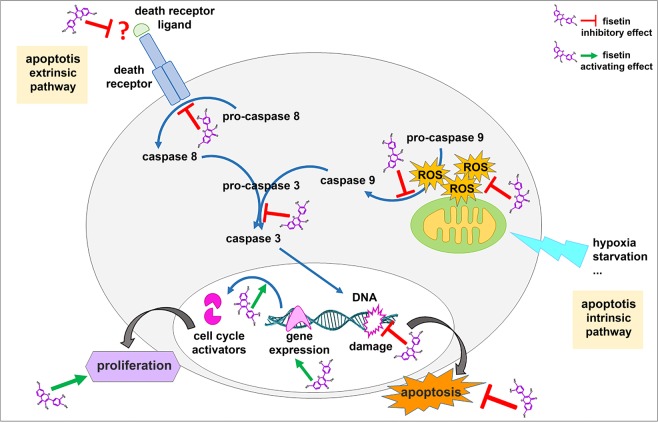
Schematic representation of fisetin cardioprotective effects on neonatal rat cardiomyocytes. Fisetin protects cells from apoptosis by targeting both intrinsic and extrinsic apoptotic pathways. Fisetin also enhances proliferation by activating expression of cell cycle activators.

Fisetin has the advantage of being non-toxic and cheap due to its wide availability as a natural product in a huge variety of fruits and vegetables. Since IRI is a major hurdle to cardiac recovery following MI, we also investigated fisetin’s protective properties against IRI for mediating cardiac recovery. We consequently validated its cardioprotective effects in mammalian cardiac cells subjected to an *in vitro* model of MI/IRI. In our model, cells were cultured under hypoxia and starvation to mimic MI, then exposed to normal conditions to mimic reoxygenation before their analysis. Fisetin was supplied to the cells during the whole reoxygenation phase to simulate its application in the clinical setting. Results indicated that fisetin clearly enhanced survival of cardiomyocytes subjected to MI/IRI. The drug was able to protect cells from apoptosis, decreased ROS generation, inhibited caspases activation and protected from DNA damage (Fig. 8).

The increased viability of cardiomyocytes in the presence of fisetin was mediated by the antiapoptotic effect of the drug. Fisetin inhibited expression of caspase 9, the initiator caspase specific of the intrinsic apoptotic pathway typically induced by hypoxia, starvation or oxidative stress, which are major consequences of HS/R. In view of the effect of fisetin on caspase 9 and ROS production, a deeper study of mitochondrial function following fisetin treatment is relevant to dissect the molecular pathways leading to fisetin’s anti-apoptotic effect. In addition, MI and IRI are also associated with elevated plasma level of TNFα^56^, one of the main activators of the extrinsic apoptotic pathway. Since fisetin inhibited expression of caspase 8, mediator of the extrinsic apoptotic pathway, we measured expression of TNFα in H9c2 cardiomyocytes culture medium after HS (6 h and over-night) and HS/R (4 h of reoxygenation) by ELISA. Intriguingly, we found that the expression of TNFα was too low to be detected in our experimental conditions (data not shown): expression of TNFα might be visible only after a very short reoxygenation period. Alternatively, fisetin treatment may instead affect regulation of other death receptors, e.g., FAS.

Furthermore, we investigated the effect of fisetin on cardioprotection. In agreement with our findings, Shanmugam *et.al*. recently showed the cardioprotective effect of fisetin using a Langendorff isolated heart perfusion system^57^. In their paper, mice were pretreated with fisetin before heart excision and induction of ischemia on the perfused heart. They showed that fisetin pretreatment attenuated myocardial injury and blunted the oxidative stress via inhibition of glycogen synthase kinase 3*β* (GSK3*β*). Despite the differences between our two experimental models - *ex vivo* experiments and pretreatment with fisetin prior to hypoxia *vs in vitro* experiments and fisetin treatment during the reoxygenation phase to mimic medication following surgery or dissolution of the thrombotic clot – our results are concordant. Our data also suggest that fisetin may have roles beyond cardiac protection, promoting cardiomyocyte proliferation and stimulating cardiomyocyte maturation. Further studies of cyclin - CDK complexes expression, degradation, phosphorylation activity and cellular localization would allow to better understand the effect of fisetin on cell cycle and DNA repair.

Summing up, we showed that fisetin protects cardiomyocytes from oxidative damage, and that further research could enable the establishment of protocols for myocardial regeneration. These findings demonstrate the value of fisetin as a candidate drug for the repositioning in MI treatment, by inhibiting ischemic damage following MI and overcoming IRI.

## Methods

Brief protocol descriptions can be found hereunder. Detailed methods are described in the Supplementary Material Supplementary Methods.

### Prediction of candidate drugs for repositioning

Candidate compounds were identified through the integrated matching of zebrafish heart regeneration expression signatures against expression signatures obtained from drug-treated cell lines in the Connectivity Map database (CMap, build 2), which contains more than 7 K expression profiles representing more than 1.3 K compounds^58^. Before implementing that procedure, we mapped gene sequences from zebrafish to humans^59^ and we developed an algorithm for matching multiple regeneration signatures to CMap drug signatures. Our prediction pipeline provided a statistically-ranked, integrated list of compounds predicted to have positive, pro-regeneration potential (Supplementary Methods).

### Chemicals

Fisetin (3,3′,4′,7-Tetrahydroxyflavone, F4043) was purchased from Sigma-Aldrich (St Louis, MO, USA). Drug was dissolved in DMSO to a stock concentration of 100 mM. The final DMSO concentration in cell culture medium never exceeded 0.1%^60^.

### Cell culture

Neonatal rat H9c2 cardiac cells (ATCC® CRL-1446™) were purchased from ATCC (Rockville, MD, USA), routinely cultured at 37 °C 5%CO_2_ in Dulbecco’s Modified Eagle’s Medium DMEM (ATCC® 30–2002™) supplemented with 10% Fetal Bovine Serum FBS (ATCC® 30–2020™) and 4 mM UltraGlutamine I (LOBE17–605E/U1, Westburg), and regularly tested for mycoplasma contamination. When sub-confluent, cells were detached with 1X Trypsin-EDTA Solution (ATCC® 30–2101™) to be subcultured.

### Hypoxia/starvation – reoxygenation and drug treatment

Cells were cultured in normal conditions (20%O_2_/5%CO_2_ at 37 °C in DMEM 10%FBS) during 24 h. Hypoxia/starvation was then simulated by culturing the cells in serum free DMEM in a hypoxia incubator (0.5%O_2_, 5%CO_2_) for 24 h^23^. To mimic reoxygenation, cells were subsequently exposed to normal conditions (20%O_2_ and DMEM 10%FBS) for 24 h^23,24^. Fisetin, at 15 μM, was applied during the whole reoxygenation period. Control cells were treated with DMSO alone (final concentration of 0.025%) as vehicle control. Cells were harvested and analyzed at the end of the reoxygenation phase.

In the cell survival assay, a range of 10 different drug concentrations was tested (from 0 to 100 μM). For the DNA damage experiment, the drug was applied during the whole hypoxia/starvation period after which cells were immediately collected and analyzed. Normoxia control was performed by culturing the cells in normal conditions during the whole experiment.

### Cell survival assay

Cell survival was assessed using the CyQuant Direct Cell Proliferation Assay Kit (C35011) from Molecular Probes (OR, USA) according to the manufacturer’s protocol (Supplementary Methods).

### Cell proliferation assay

Experiments were performed by flow cytometry. Cells were harvested following drug treatment, washed, then stained with Ki67 antibody as described in Supplementary Methods.

### Gene expression measurements

From 1 to 5 × 10^6^ H9c2 cells were harvested following drug treatment and washed in 1x PBS before RNA extraction using TRI Reagent^®^ (Sigma-Aldrich). Subsequent RNA isolation, qRT-PCR techniques and data analysis are described in Supplementary Methods.

### Cell death assay

Cell death was assessed by annexin V/PI staining followed by flow cytometry as described in Supplementary Methods.

### Measurement of reactive oxygen species

Cells were harvested, washed in pre-warmed to 37 °C 1x PBS, and incubated for 30 min at 37 °C in the dark in a solution of pre-warmed 1x PBS - 10 μM Dihydrorhodamine 123 (D23806, Invitrogen) at a cell density of 10^6^ cells/mL. Cells were further washed in pre-warmed 1x PBS, resuspended in pre-warmed 1x PBS containing 1 μg/mL propidium iodide (P3566, Invitrogen) and analyzed by FACS on a BDFACS Canto™ Flow cytometer (BD Biosciences). We reported the mean intensity of three independent experiments acquired and analyzed using DIVA (BD Biosciences) and GraphPad Prism 7 (GraphPad).

### Caspases activity assay

Caspases activity assays were performed using the CaspGLOW™ Fluorescein Active Caspase Staining Kits (88–7004, 88–7005 and 88–7006 for caspase-3, 8 and 9 respectively, eBioscience), following the manufacturer’s protocol. Data were acquired and analyzed by FACS on a BDFACS Canto™ Flow cytometer (BD Biosciences) using DIVA (BD Biosciences) and GraphPad Prism 7 (GraphPad). We reported the mean intensity of three independent experiments.

### Measurement of DNA damage

Drugs were applied during the whole hypoxia/starvation period. Cells were harvested immediately after and subjected to flow cytometry analysis. Cells were washed in HBSS, 2% FBS, 10 mM HEPES pH7.4, stained with 1 µg/ml of LIVE/DEAD® Fixable Near-IR Dead Cell Stain Kit (L34975, Invitrogen) then fixed and permeabilized in BD Cytofix/Cytoperm™ solution (554722, BD Biosciences). Cells were then washed in BD Perm/Wash™ buffer (554723, BD Biosciences) and stained for 30 min in the dark with the FITC- Mouse Anti-8 Hydroxyguanosine antibody [15A3] (ab183393, Abcam) used at 1 µL/10^6^ cells or the FITC- Mouse IGg2b antibody (21275533, Immunotools) as isotype control. We reported the mean intensity of three independent experiments. Data acquisition and analysis were performed using DIVA (BD Biosciences) and GraphPad Prism 7 (GraphPad).

## Supplementary information

SUPPLEMENTARY MATERIAL.

MIQE.

ADDITIONAL MIQE A&B.
