# Supplementary material for: Fisetin protects against cardiac cell death through reduction of ROS production and caspases activity
Source: Sci Rep. 2020 Feb 19;10:2896. doi: 10.1038/s41598-020-59894-4 (PMC7031222; doi:10.1038/s41598-020-59894-4)
Supplement: Supplementary file 1 — SUPPLEMENTARY MATERIAL. [file 41598_2020_59894_MOESM1_ESM.pdf]

Fisetin protects against cardiac cell death through reduction of ROS production and caspases activity.

Sophie Rodius[1], Niek de Klein[2,3], Céline Jeanty[1], Héctor Sánchez-Iranzo[4], Isaac Crespo[5], Mark Ibberson[5], Ioannis Xenarios[5,6,7], Gunnar Dittmar[1], Nadia Mercader[4,8], Simone P. Niclou[9], Francisco Azuaje[1,10]

SUPPLEMENTARY MATERIAL

SUPPLEMENTARY METHODS

Prediction of candidate drugs for repositioning

Candidate compounds were identified by the integrated matching of zebrafish heart regeneration expression signatures against expression signatures obtained from drug-treated cell lines in the Connectivity Map database (CMap, build 2), which contains more than 7K expression profiles representing more than 1.3K compounds[1]. Before implementing that procedure, we mapped gene sequences from zebrafish to humans[2]. Four different resources were used to map Zebrafish affymetrix probe IDs to Human affymetrix probe IDs in the following order, only trying subsequent steps if the one before did not work: 1. Convert the Zebrafish Affymetrix ID to Zebrafish probe symbol, search the gene on ZFIN and extract the Human homolog entrez ID[3]; 2. Convert Zebrafish Affymetrix ID to Zebrafish ensemble ID or probe symbol, use Biomart to convert it to a Human homolog entrez ID[4]; 3. Convert the Zebrafish Affymetrix ID to zebrafish probe symbol, search with HomoloGene for Human homolog entrez ID[5,6]; 4 or finally, convert Zebrafish Affymetrix ID to Zebrafish uniprot ID, use that protein sequence to BLAST against Human database of proteins, and select the entrez ID for the most significant hit (if $p < 0.05$)[7-9]. Finally, we converted the Human entrez ID to human Affymetrix ID with affy[10] and ArrayExpress[11]. Originally, the CMap was designed to provide users with a single list of ranked compounds, whose expression signatures are similar (or dissimilar) to that encoded in a single (user-defined) query signature. Therefore, here we had to develop an algorithm for matching multiple regeneration signatures to CMap drug signatures. Our prediction pipeline provided a statistically-ranked, integrated list of compounds predicted to have positive, pro-regeneration potential. First, we identified regeneration signatures by selecting differentially expressed genes between different time points during the regeneration process (from 4 hours to 90 days post-injury) using the gene expression data that we reported in[12]. This analysis was done with lmFit and eBayes from the R limma package[13]. At each (regeneration) time point in relation to 4 hours post-injury, we identified differentially expressed genes with an absolute fold change > 2 and Bonferroni corrected p-value < 0.05, and included them in the time-specific signatures. These signatures were then used independently to search the CMap. For each regeneration signature, CMap compounds were ranked on the basis of the similarity between their expression profiles and the regeneration signatures (using CMap-generated p-values of significance). Next, for each drug, we calculated a rank product of the drug's signature matchings to the regeneration signatures[14], and used this score to compute integrated p-values reflecting the overall drug's signature similarity across time points. These p-values were estimated using a heuristic method proposed by Eisinga et al.[15]. The code that implements this strategy is available at https://gitlab.com/biomodlih/drugFinder.

Cell survival assay

Cell survival was assessed using the CyQuant Direct Cell Proliferation Assay Kit (C35011) from Molecular Probes (OR, USA). Cells were seeded in 96 well-plates at a density of 4000 cells/well, cultured in normal condition during 24h, and then subjected to 24h of hypoxia/starvation followed by 24h of reoxygenation in the presence of the drug. Triplicate wells were treated with 5 to 100μM of fisetin. Control cells were treated with vehicle alone (DMSO at 0.1%). In normoxia or HS experiments, cells were cultured in normal conditions for 24h, then treated with the drug for 24h in normal conditions for cells in normoxia or in serum free DMEM at 0.5% 0_2 for HS experiment, followed by their analysis. The CyQuant assay was performed following the manufacturer's instructions. Fluorescence was measured on a FLUOstar OPTIMA Microplate Reader (BMG LABTECH) at 492nm excitation and 520nm emission. Results are reported as the mean intensity of three independent experiments using GraphPad Prism 7 (GraphPad).

Cell proliferation assay

Experiments were performed by flow cytometry. Cells were harvested following drug treatment, washed and resuspended in ice-cold Hank's balanced salt solution (HBSS) containing 2% FBS and 10mM HEPES pH7.4. Cells were stained with 1µg/ml of LIVE/DEAD® Fixable Near-IR Dead Cell Stain Kit (L34975, Invitrogen), then fixed and permeabilized with cold 80% ethanol. Cells were rehydrated in HBSS, 2% FBS, 10mM HEPES pH7.4 and proliferation was assessed by staining the cells for 30min in the dark with a PE-anti Ki67 antibody (556027, BD Pharmingen) used at 2µL/10^5 cells. The PE-Mouse IgG1 k antibody (555749, BD Pharmingen) was used as isotype control. Cells were incubated for 15min in the dark in a 5µg/mL DAPI solution (DAPI dilactate, D3571, Invitrogen) before acquisition. Data were acquired on a fluorescence-activated cell sorting (FACS) Aria™ SORP cytometer (BD Biosciences). Data acquisition and analysis were performed using the DIVA (BD Biosciences) and GraphPad Prism 7 (GraphPad). We report the mean fluorescence intensity of three independent experiments.

Gene expression measurements

From 1 to 5 x 10^6 H9C2 cells were harvested following drug treatment, washed in 1x PBS and RNA was extracted from cells using TRI Reagent® (Sigma-Aldrich). RNA isolated in the aqueous phase with a Phase lock gel-Heavy (5 Prime) was precipitated with 100% isopropanol and purified using RNeasy® Mini kit combined with an on-column DNase treatment (Qiagen). RNA was quantified using Nanodrop™ (Thermo Fisher Scientific) and integrity was verified with Bioanalyzer (Agilent). For RT-PCR, 1µg RNA was reverse-transcribed into cDNA using Superscript III™ (Invitrogen) following manufacturer's instructions. Then, the qRT-PCR was realized in 384-well plates using SsoAdvanced™ Universal SYBR® Green Supermix (Bio-Rad) and the Viia™7 real-time PCR system (Applied Biosystems™). Quantitative PCR data analysis was done using qbase+ software, version 3.1 (Biogazelle, Zwijnaarde, Belgium - www.qbaseplus.com). Normalized gene expression levels were calculated via the delta-delta Cq method with *Eef1a1* and *Rpl4* as reference genes and taking into account the calculated amplification efficiency for each primers pair. The mean comparisons to normoxia condition were performed with four independent experiments. Statistical significance was determined using an ANOVA one-way corrected for multiple testing with a Tukey-Kramer as post-test (corrected p-value < 0.05) - see Supplementary Material Supplementary Table for MIQE checklist and qRT-PCR experiment details.

Cell death assay

Cell death was assessed by annexin V / PI staining followed by flow cytometry. Cells were trypsinized, washed in HBSS, 2% FBS, 10mM HEPES pH7.4 and stained for 30min in the dark with annexin V – APC conjugated antibody (31490016, Immunotools) diluted at 2µL/10^5 cells in annexin V binding buffer (10mM HEPES pH7.4, 140mM NaCl, 2,5mM $CaCl_2$). Cells were washed,

resuspended in annexin V binding buffer containing 1μg/mL propidium iodide (P3566, Invitrogen) and subjected to FACS analysis on a BDFACS Canto™ Flow cytometer (BD Biosciences). We report the mean intensity of three independent experiments. Data acquisition and analysis were performed with DIVA (BD Biosciences) and GraphPad Prism 7 (GraphPad).

SUPPLEMENTARY REFERENCES

1 Lamb, J. *et al.* The Connectivity Map: using gene-expression signatures to connect small molecules, genes, and disease. *Science* **313**, 1929-1935, doi:10.1126/science.1132939 (2006).

2 de Klein, N., Ibberson, M., Crespo, I., Rodius, S. & Azuaje, F. A gene mapping bottleneck in the translational route from zebrafish to human. *Front Genet* **5**, 470, doi:10.3389/fgene.2014.00470 (2014).

3 Bradford, Y. *et al.* ZFIN: enhancements and updates to the Zebrafish Model Organism Database. *Nucleic acids research* **39**, D822-829, doi:10.1093/nar/gkq1077 (2011).

4 Kasprzyk, A. BioMart: driving a paradigm change in biological data management. *Database : the journal of biological databases and curation* **2011**, bar049, doi:10.1093/database/bar049 (2011).

5 Wheeler, D. L. *et al.* Database resources of the National Center for Biotechnology Information. *Nucleic acids research* **29**, 11-16 (2001).

6 Acland, A. *et al.* Database resources of the National Center for Biotechnology Information. *Nucleic acids research* **42** (2014).

7 Altschul, S. F., Gish, W., Miller, W., Myers, E. W. & Lipman, D. J. Basic local alignment search tool. *Journal of molecular biology* **215**, 403-410, doi:10.1016/S0022-2836(05)80360-2 (1990).

8 Altschul, S. F. *et al.* Gapped BLAST and PSI-BLAST: a new generation of protein database search programs. *Nucleic acids research* **25**, 3389-3402 (1997).

9 Ye, J., McGinnis, S. & Madden, T. L. BLAST: improvements for better sequence analysis. *Nucleic acids research* **34**, W6-9, doi:10.1093/nar/gkl164 (2006).

10 Gautier, L., Cope, L., Bolstad, B. M. & Irizarry, R. A. affy--analysis of Affymetrix GeneChip data at the probe level. *Bioinformatics* **20**, 307-315, doi:10.1093/bioinformatics/btg405 (2004).

11 Brazma, A. *et al.* ArrayExpress--a public repository for microarray gene expression data at the EBI. *Nucleic acids research* **31**, 68-71 (2003).

12 Rodius, S. *et al.* Analysis of the dynamic co-expression network of heart regeneration in the zebrafish. *Sci Rep* **6**, 26822, doi:10.1038/srep26822 (2016).

13 Smyth, G. K. Linear models and empirical bayes methods for assessing differential expression in microarray experiments. *Statistical applications in genetics and molecular biology* **3**, Article3, doi:10.2202/1544-6115.1027 (2004).

14 Breitling, R., Armengaud, P., Amtmann, A. & Herzyk, P. Rank products: a simple, yet powerful, new method to detect differentially regulated genes in replicated microarray experiments. *FEBS Lett* **573**, 83-92, doi:10.1016/j.febslet.2004.07.055 (2004).

15 Eisinga, R., Breitling, R. & Heskes, T. The exact probability distribution of the rank product statistics for replicated experiments. *FEBS Lett* **587**, 677-682, doi:10.1016/j.febslet.2013.01.037 (2013).
