# Supplementary material for: Fisetin protects against cardiac cell death through reduction of ROS production and caspases activity
Source: Sci Rep. 2020 Feb 19;10:2896. doi: 10.1038/s41598-020-59894-4 (PMC7031222; doi:10.1038/s41598-020-59894-4)
Supplement: Supplementary file 2 — MIQE. [file 41598_2020_59894_MOESM2_ESM.pdf]

ITEM TO CHECK	IMPORTANCE	CHECKLIST
**EXPERIMENTAL DESIGN**		
Definition of experimental and control groups	**E**	Rat H9c2 cardiac cells were cultured under normoxia during 48h (Normoxia). In parallel, the cells were subjected to Hypoxia/starvation (24h), then reoxygenated and treated with DMSO as vehicle control (HS/R) or with 15µM fisetin (HS/R + F) during 24h.
Number within each group	**E**	n=4
Assay carried out by core lab or investigator's lab?	D	
Acknowledgement of authors' contributions	D	
**SAMPLE**		
Description	**E**	Rat H9c2 cardiac cells (ATCC® CRL-1446™) were purchased from ATCC (Rockville, MD, USA).
Volume/mass of sample processed	D	From 1 to 5 x 10^6 H9C2 cells were harvested following drug treatment.
Microdissection or macrodissection	**E**	N/A
Processing procedure	**E**	Cells were washed, counted in PBS (without Ca^{++} and Mg^{++}), centrifuged, resuspended in 1mL TRI Reagent®, snap-frozen and then stored at -80°C.
If frozen - how and how quickly?	**E**	Samples were snap-frozen in TRI Reagent® and stored at -80°C.
If fixed - with what, how quickly?	**E**	N/A
Sample storage conditions and duration (especially for FFPE samples)	**E**	Samples were stored in TRI Reagent® at -80°C until RNA extraction.
**NUCLEIC ACID EXTRACTION**		
Procedure and/or instrumentation	**E**	Total RNA was extracted from 1 to 5 x 10^6 cells with a TRI Reagent® (Sigma-Aldrich) isolation protocol. Aqueous phase was isolated with Phase lock gel-Heavy (5 Prime, Gaithersburg, MD). Total RNA was precipitated with 100% isopropanol and purified with a RNeasy® Mini kit combined with an on-column DNase treatment following the manufacturer's instructions(Qiagen, Valencia, CA).
Name of kit and details of any modifications	**E**	TRI Reagent® - RNeasy® Mini kit combined with an on-column DNase treatment following the manufacturer's instructions.
Source of additional reagents used	D	Chloroform (Merck); Ispropanol (Merk); Ethanol (Merck); Nuclease free water (Life Technologies)
Details of DNase or RNAse treatment	**E**	RNeasy® Mini kit combined with an on-column DNase treatment following the manufacturer's instructions.
Contamination assessment (DNA or RNA)	**E**	DNase treatment + Bioanalyzer + primers flanking intron + Negative control (RT-PCR & qPCR)
Nucleic acid quantification	**E**	Nanodrop
Instrument and method	**E**	Nanodrop
Purity (A260/A280)	D	All RNA sample : Purity (A260/A280) ≈ 2
Yield	D	N/A
RNA integrity method/instrument	**E**	Bioanalyzer
RIN/RQI or Cq of 3' and 5' transcripts	**E**	All RNA sample : RIN ≥ 9
Electrophoresis traces	D	N/A
Inhibition testing (Cq dilutions, spike or other)	**E**	Inhibition tested with a serial dilution method to monitor linearity of Cq values.
**REVERSE TRANSCRIPTION**		
Complete reaction conditions	**E**	1µg of RNA were reverse transcribed into cDNA using the SuperScript III (Invitrogen, Carlsbad, CA) reverse transcriptase with the following protocol: RNAs were mixed with random primers, oligo (dT)12-18 and dNTPs in a total volume of 13µl. Samples were heated to 65°C for 5 min and incubated on ice for at least 1min. Then the 5X RT buffer, DTT, RNaseOUT and SuperScript III was added to a total volume of 20µl. RT was allowed at 50°C for 60 min. and was followed by enzyme inactivation at 70°C for 15 min. Final concentrations were: 100ng of oligo(dT)12-18, 50ng of random primers, 0.5mM dNTPs, 50mM Tris-HCl, 75mM KCl, 3mM MgCl2, 5mM DTT, 40U of RNaseOUT and 200U of SuperScript III. To remove RNA complementary to the cDNA, 2U of *E.coli* RNaseH was added and incubated at 37°C for 20 minutes. In each RT-PCR a no template control (no RNA in RT) were performed.
Amount of RNA and reaction volume	**E**	1µg RNA / 20µl reaction volume
Priming oligonucleotide (if using GSP) and concentration	**E**	Random primers : 2.5ng/µl Oligo$(dT)_{12-18}$: 5ng/µl (final concentration)
Reverse transcriptase and concentration	**E**	SuperScript III (Invitrogen) : 10U/µl (final concentration)
Temperature and time	**E**	RNAs were mixed with random primers, oligo (dT)12-18 and dNTPs in a total volume of 13µl. Samples were heated to 65°C for 5 min and incubated on ice for at least 1min. Then the 5X RT buffer, DTT, RNaseOUT and SuperScript III was added to a total volume of 20µl. RT was allowed at 50°C for 60 min. and was followed by enzyme inactivation at 70°C for 15 min. To remove RNA complementary to the cDNA, 2U of *E.coli* RNaseH was added and incubated at 37°C for 20 minutes.
Manufacturer of reagents and catalogue numbers	D	Life Technologies : SuperScript III (Cat.18080-085), Oligo$(dT)_{12-18}$ primer (18418-012), Random primers (Cat. 48190-011), 10mM dNTP Mix (18427-013), RNaseOUT 40U/µl (10777-019), *E.coli* RNaseH (AM2293)
Cqs with and without RT	D*	N/A - DNase treatment + primers flanking intron + Melt Curve
Storage conditions of cDNA	D	-20°C
**qPCR TARGET INFORMATION**		
If multiplex, efficiency and LOD of each assay.	**E**	N/A
Sequence accession number	**E**	see additional MIQE-A
Location of amplicon	D	see additional MIQE-A
Amplicon length	**E**	see additional MIQE-A
In silico specificity screen (BLAST, etc)	**E**	Beacon Designer Pro 8.10 software (Premier Biosoft) + NCBI BLAST tool
Pseudogenes, retropseudogenes or other homologs?	D	
Sequence alignment	D	
Secondary structure analysis of amplicon	D	
Location of each primer by exon or intron (if applicable)	**E**	see additional MIQE-A
What splice variants are targeted?	E	see additional MIQE-A cf. Accession number
**qPCR OLIGONUCLEOTIDES**		
Primer sequences	**E**	see additional MIQE-A
RTPrimerDB Identification Number	D	N/A
Probe sequences	D**	N/A
Location and identity of any modifications	**E**	N/A
Manufacturer of oligonucleotides	D	EUROGENTEC (Seraing, Belgium)
Purification method	D	RP-Cartridge - Gold
**qPCR PROTOCOL**		
Complete reaction conditions	**E**	cDNAs obtained from RT-PCR of RNA were diluted 10-fold and 2µL were mixed with SsoAdvanced™ Universal SYBR® Green Supermix (Bio-Rad, Nazareth, Belgium) to a final volume of 10µL containing 300nM of each primer. Amplification was carried out in the Viia™7 real-time PCR 384-well system (Applied Biosystems™) under the following conditions: heating for 3 minutes at 95°C, 40 cycles of denaturation for 15 seconds at 95°C, followed by an annealing/extension for 1 min. After each run, a melting curve was performed with continuous temperature ramping at 0.05°C/s from 55°C to 95°C. A negative control without cDNA template was run in every assay and measures were performed in triplicates.
Reaction volume and amount of cDNA/DNA	**E**	2µl-cDNA diluted 10 fold / 10µl reaction volume
Primer, (probe), Mg++ and dNTP concentrations	**E**	300nM of each primer + SsoAdvanced™ Universal SYBR® Green Supermix
Polymerase identity and concentration	**E**	Sso7d fusion polymerase
Buffer/kit identity and manufacturer	**E**	SsoAdvanced™ Universal SYBR® Green Supermix (Biorad, Nazareth, Belgium) (Cat. 172-5275)
Exact chemical constitution of the buffer	D	
Additives (SYBR Green I, DMSO, etc.)	**E**	N/A
Manufacturer of plates/tubes and catalog number	D	
Complete thermocycling parameters	**E**	Heating for 3 minutes at 95°C, 40 cycles : denaturation for 15 seconds at 95°C, followed by an annealing/extension for 1 min.<br>After each run, a melting curve was performed with continuous temperature ramping at 0.05°C/s from 55°C to 95°C.
Reaction setup (manual/robotic)	D	
Manufacturer of qPCR instrument	**E**	CFX96 thermal cycler (BioRad) / Viia™7 real-time PCR 384-well system (Applied Biosystems™)
**qPCR VALIDATION**		
Evidence of optimisation (from gradients)	D	Temperature gradient performed in the CFX96 thermal cycler (BioRad) qPCR efficiency and experiment performed in the Viia™7 real-time PCR 384-well system (Applied Biosystems™)
Specificity (gel, sequence, melt, or digest)	**E**	Gene-specific amplification confirmed by a single band in 4% E-Gel® (Life technologies).<br>Melt Curve analysis performed in each assay.<br>No template control (NTC) (no cDNA in qPCR) ran for each gene to verify unspecific amplification and/or primers dimerization.
For SYBR Green I, Cq of the NTC	**E**	No amplification signal detected
Standard curves with slope and y-intercept	**E**	see additional MIQE-A
PCR efficiency calculated from slope	**E**	see additional MIQE-A
Confidence interval for PCR efficiency or standard error	D	
r2 of standard curve	**E**	see additional MIQE-A
Linear dynamic range	**E**	see additional MIQE-A
Cq variation at lower limit	**E**	see additional MIQE-A
Confidence intervals throughout range	D	
Evidence for limit of detection	**E**	see additional MIQE-A
If multiplex, efficiency and LOD of each assay.	**E**	N/A
**DATA ANALYSIS**		
qPCR analysis program (source, version)	**E**	QuantStudioTM Real-Time PCR Software v1.3
Cq method determination	**E**	The automatic threshold was used with the QuantStudio™ Real-Time PCR Software v1.3 program. An analysis setting for the Baseline Threshold algorithm in which the software calculates the baseline start and end cycles and the threshold in the amplification plot. The software uses the baseline and threshold to calculate the threshold cycle (Cq).
Outlier identification and disposition	**E**	Bad replicates and measurements below LOD were discarded
Results of NTCs	**E**	No amplification signal detected
Justification of number and choice of reference genes	**E**	6 reference genes were tested in geNorm. Data normalization was carried out against two reference genes; Eef1a1 and Rpl4. see additional MIQE-B
Description of normalisation method	**E**	Normalized gene expression levels were calculated via the delta-delta Cq method with Eef1a1 and Rpl4 as reference genes and taking into account the calculated amplification efficiency for each primers pair. The mean comparisons to normoxia condition were performed with four independent experiments.
Number and concordance of biological replicates	D	
Number and stage (RT or qPCR) of technical replicates	**E**	qPCR reactions were performed in triplicates
Repeatability (intra-assay variation)	**E**	For each sample, standard deviation (SD) for the Cq variation between replicates has been used to express intra-assay variation.<br>Instrument and liquid handling variations were shown to be minimal.
Reproducibility (inter-assay variation, %CV)	D	
Power analysis	D	
Statistical methods for result significance	**E**	Statistical significance was determined using an ANOVA one-way corrected for multiple testing with a Tukey-Kramer as post-test (corrected p-value < 0.05)
Software (source, version)	E	qbase+ software, version 3.1 (Biogazelle, Zwijnaarde, Belgium - www.qbaseplus.com)
Cq or raw data submission using RDML	**D**	

Table 1. MIQE checklist for authors, reviewers and editors. All essential information (E) must be submitted with the manuscript. Desirable information (D) should be submitted if available. If using primers obtained from RTPrimerDB, information on qPCR target, oligonucleotides, protocols and validation is available from that source.

*: Assessing the absence of DNA using a no RT assay is essential when first extracting RNA. Once the sample has been validated as RDNA-free, inclusion of a no-RT control is desirable, but no longer essential.

**: Disclosure of the probe sequence is highly desirable and strongly encouraged. However, since not all commercial pre-designed assay vendors provide this information, it cannot be an essential requirement. Use of such assays is advised against.
