# Supplementary material for: Fisetin protects against cardiac cell death through reduction of ROS production and caspases activity
Source: Sci Rep. 2020 Feb 19;10:2896. doi: 10.1038/s41598-020-59894-4 (PMC7031222; doi:10.1038/s41598-020-59894-4)
Supplement: Supplementary file 3 — ADDITIONAL MIQE A&B. [file 41598_2020_59894_MOESM3_ESM.pdf]

A- MIQE qPCR primers information

Gene Name	Accession number	Foward primer sequence (5'>3')	Reverse primer sequence (5'>3')	Primers location	Amplicon (pb)	Standard curves	PCR efficiency (%)	r2
**Actc1**	NM_019183	CATTCACGAAACTACTTA	GAGCAATAATCTTAATCTTC	F:E 6 - R:E 6-7 (Intron 506pb)	176	y= 19.736 x -3.294	101,18	0,997
**Actn2**	NM_001170325	GCAGTCCATCCTAGCCATCC	GGCGCTCATTAGCATGTTGG	F:E 15 - R:E 16 (Intron 1386pb)	197	y= 23.578 x -3.232	103,89	0,980
**Ccnd2**	NM_022267	ACCGATGTGGATTGTCTC	TTGACGGAACTGCTGAAG	F:E 4-5 - R:E 5 (Intron 10060pb)	78	y= 18.761 x -3.438	95,39	0,998
**Ccne1**	NM_001100821	CTTCCCGTCTTGAATTGG	CTCTCAGGTAAGTCTTCTCT	F:E 5 - R:E 6 (Intron 114pb)	76	y= 19.864 x -3.255	102,89	0,992
**Ccne2**	NM_001108656	CAGACTCTCCACAAGAAG	CAACAATTCCTAATCTCATACT	F:E 3 - R:E 4-5 (Intron 82-681pb)	118	y= 21.021 x -3.236	103,73	0,996
**Cdk1**	NM_019296	GATTCTTCGCTCGTTAAG	CCAGTTTGATTGTTCCTT	F:E 4 - R:E 5 (Intron 2467pb)	133	y= 17.156 x -3.502	93,00	0,999
**Cdk6**	NM_001191861	TCCTTCTGAAATGCTTGA	GGTTGTCCTTGTATCTCT	F:E 6-7 - R:E 7 (Intron 1296pb)	102	y= 20.396 x -3.196	105,55	0,998
**Cdkn1a**	NM_080782	GACATCTCAGGGCCGAAA	TTCTCTTGCAGAAGACCAATC	F:E 2 - R:E 3 (Intron 582pb)	83	y= 19.631 x -3.286	101,53	0,996
**Eef1a1**	NM_175838	GACAATGTAGGCTTCAAC	ATAATCACCTGAGCAGTG	F:E 5 - R:E 5-6 (Intron 89pb)	122	y= 12.026x -3.311	100,45	0,999
**Fgf2**	NM_019305	GCTATGAAGGAAGATGGA	GTAAGTGTTGTAGTTATTGGA	F:E 2 - R:E 3 (Intron 10484pb)	96	y= 22.330 x -3.297	101,03	0,967
**Foxm1**	NM_031633	CTAATCGCTACTTGACATTG	TTTCTGCTGTGATTCCAA	F:E 6 - R:E 7-8 (Intron 529pb)	89	y= 19.086 x -3.426	95,83	0,997
**Gata4**	NM_144730	AATGCGGAAGGAGGGGATT	AGGACCTGCTGGTGTCTTAG	F:E 5 - R:E 5-6 (Intron 1643pb)	76	y= 22.825 x -3.401	96,79	0,995
**Hmox1**	NM_012580	GAACTTTCAGAAGGGTCAG	GCCGTATAGATATGGTACAAG	F:E 2 - R:E 3 (Intron 826pb)	75	y= 17.497 x -3.359	98,46	0,997
**Igf1r**	NM_052807	ACGGATTGATTCTAATGTATGA	CTTCCTGTACTCCTGTCT	F:E 12 - R:E 13 (Intron 465pb)	86	y= 19.041 x -3.368	98,13	0,997
**Il6**	NM_012589	GTGAAGAACAACTTACAAGA	CATTAGGAGAGCATTGGA	F:E 4 - R:E 5 (Intron 1225pb)	147	y= 19.940 x -3.431	95,65	0,996
**Nkx2-5**	NM_053651	TTTTATCCGCGAGCCTACGG	TCTGTCTCGGCTTTGTCCAG	F:E 1 - R:E 2 (Intron 1432pb)	110	y= 26.791 x -2.932	119,30	0,930
**Rpl4**	NM_022510	AGTTGGATGAGTTGTATG	TTCAAGATTCTGCTAAGG	F:E 7 - R:E 8 (Intron 331pb)	106	y= 13.886 x -3.371	98,00	0,999
**Sirt1**	XM_006256146;XM_008772947; XM_006223877; XM_008774951; XM_008774950	TGTCAGATAAGGAAGGAA	TTTACAAATCAGGCAAGA	F:E 4 - R:E 5 (Intron 636pb)	131	y= 21.151 x -3.340	99,26	0,997
**Tgfb1**	NM_021578	AGAGATTCAAGTCAACTGTGGAG	CCAAGGTAACGCCAGGAA	F:E 2 - R:E 3 (Intron 1616pb)	76	y= 16.215 x -3.265	102,40	1,000

B- MIQE data analysis information

Target Stability

Target	Coefficient Variance	M-Value
**Eef1a1**	0,050	0,142
**Rpl4**	0,049	0,142

Average Coefficient Variance: : 0.049
Average M-Value: : 0.142

Coefficient of Variation (CV) of normalized reference gene relative quantities. A lower CV value denotes higher stability
M-value. A measure of the reference gene expression stability

Linear dynamic range	Cq variation at lower limit
from 4.00E-02 H9C2 cDNA dilution to 4.938E-04	30.561 ± 0.193
from 4.00E-02 H9C2 cDNA dilution to 2.500E-03	31.940 ± 0.140
from 4.00E-02 H9C2 cDNA dilution to 1.280E-05	35.643 ± 0.203
from 4.00E-02 H9C2 cDNA dilution to 4.938E-04	30.536 ± 0.061
from 4.00E-02 H9C2 cDNA dilution to 2.500E-03	29.474 ± 0.121
from 4.00E-02 H9C2 cDNA dilution to 3.200E-04	29.387 ± 0.199
from 4.00E-02 H9C2 cDNA dilution to 1.646E-04	32.532 ± 0.160
from 4.00E-02 H9C2 cDNA dilution to 1.646E-04	32.002 ± 0.361
from 4.00E-02 H9C2 cDNA dilution to 3.999E-06	29.849 ± 0.300
from 4.00E-02 H9C2 cDNA dilution to 1.250E-03	31.633 ± 0.451
from 4.00E-02 H9C2 cDNA dilution to 1.646E-04	32.004 ± 0.192
from 4.00E-02 H9C2 cDNA dilution to 2.500E-03	31.760 ± 0.050
from 4.00E-02 H9C2 cDNA dilution to 3.91E-05	32.121 ± 0.241
from 4.00E-02 H9C2 cDNA dilution to 1.646E-04	31.622 ± 0.164
from 4.00E-02 H9C2 cDNA dilution to 1.646E-04	32.921 ± 0.329
from 4.00E-02 H9C2 cDNA dilution to 5.000E-03	33.785 ± 0.261
from 4.00E-02 H9C2 cDNA dilution to 1.220E-06	34.012 ± 0.035
from 4.00E-02 H9C2 cDNA dilution to 1.481E-04	30.673 ± 0.003
from 4.00E-02 H9C2 cDNA dilution to 1.56E-04	28.65 ± 0.09
